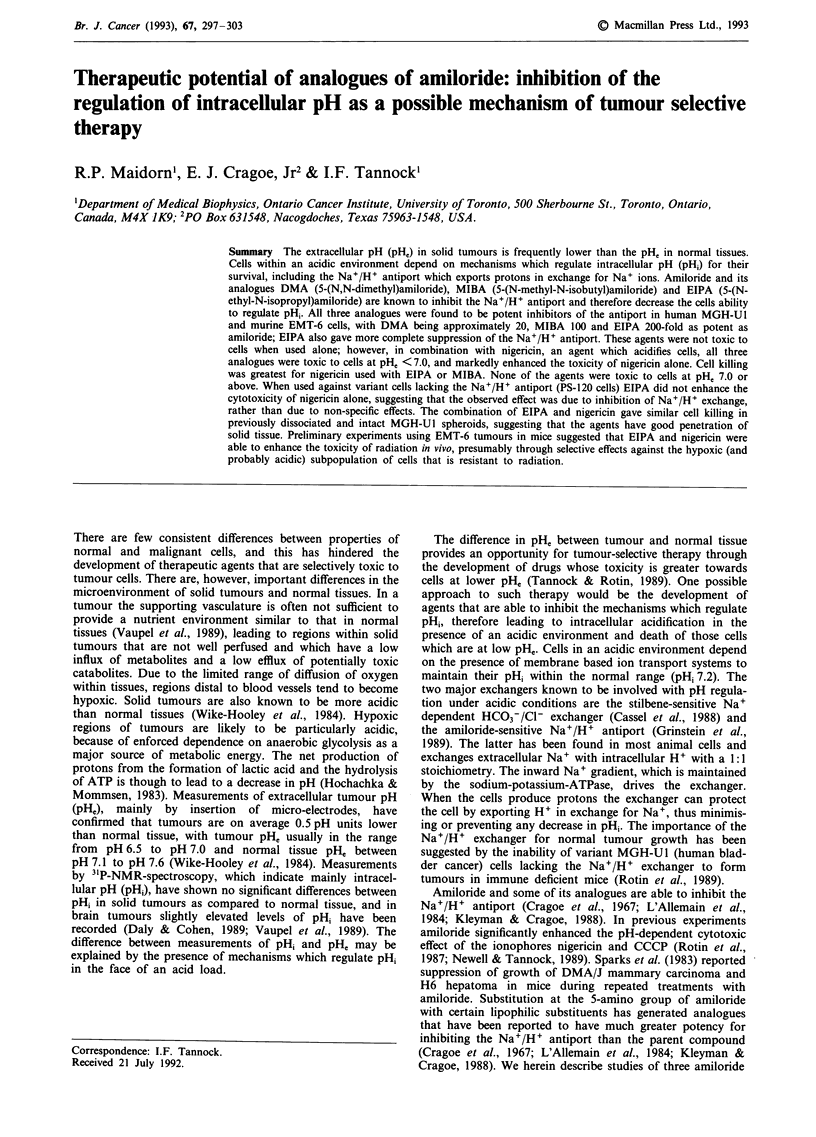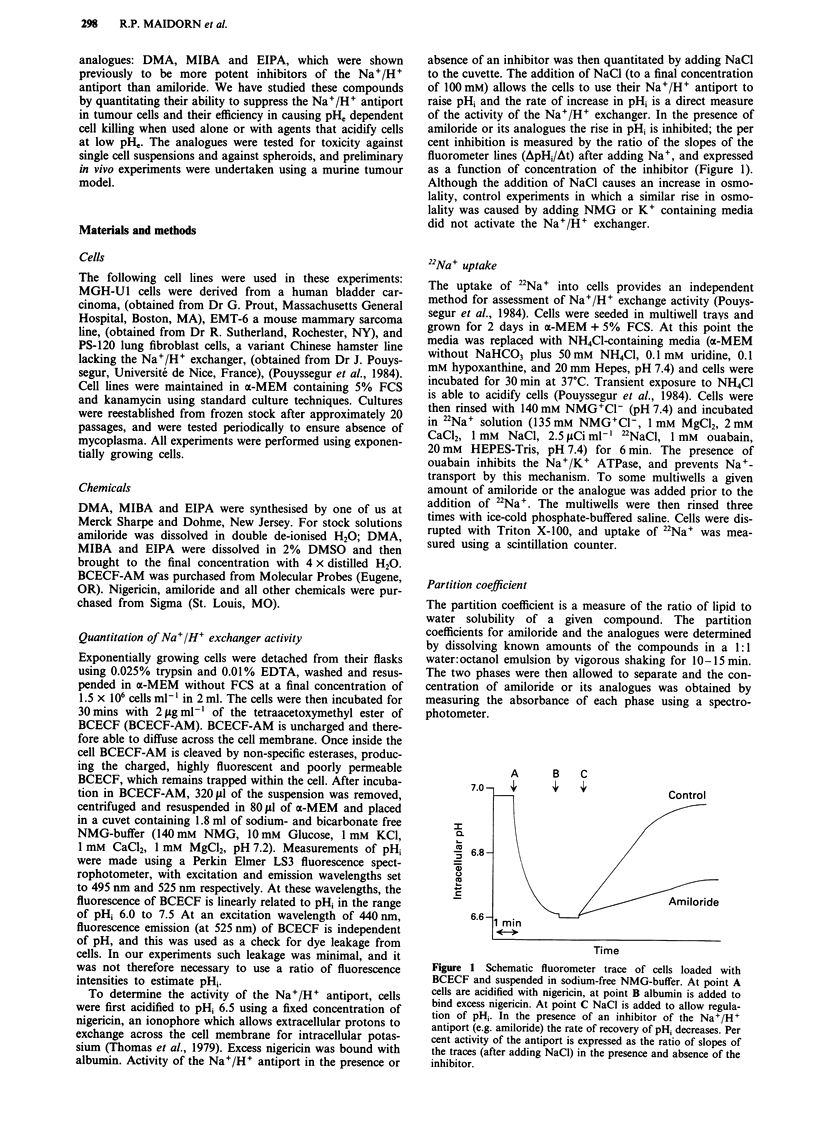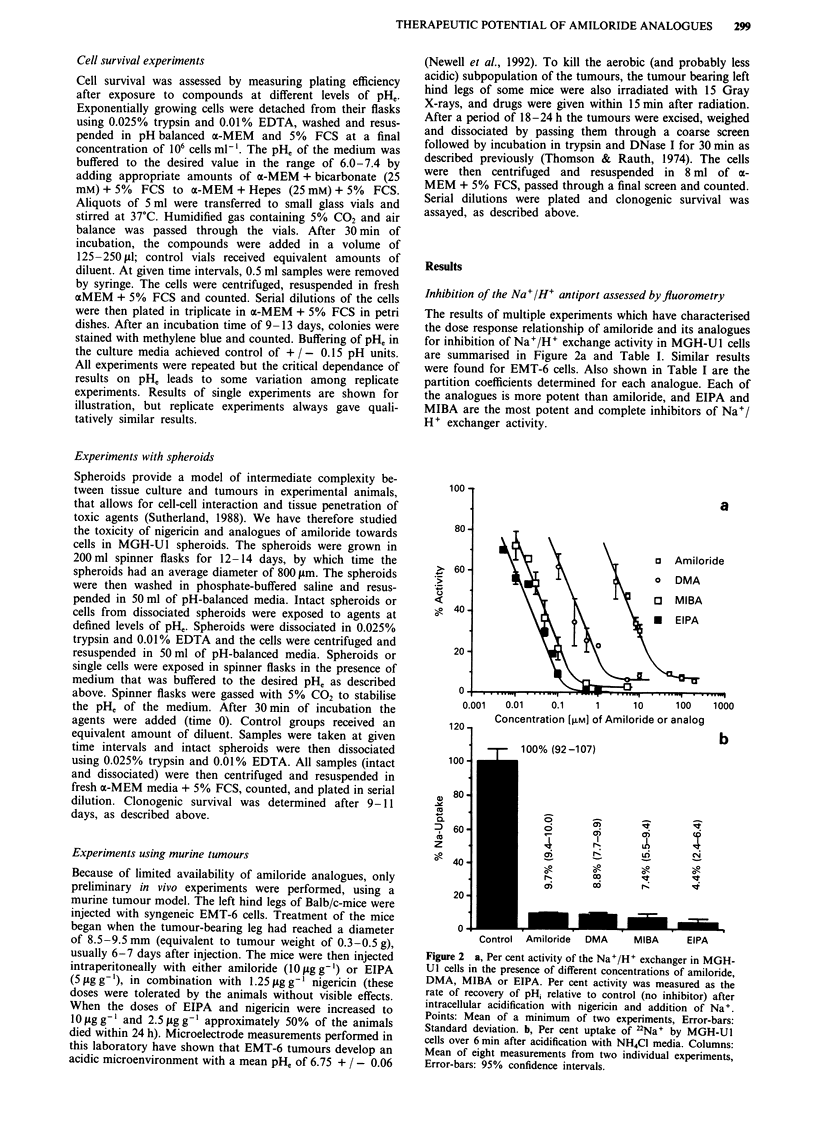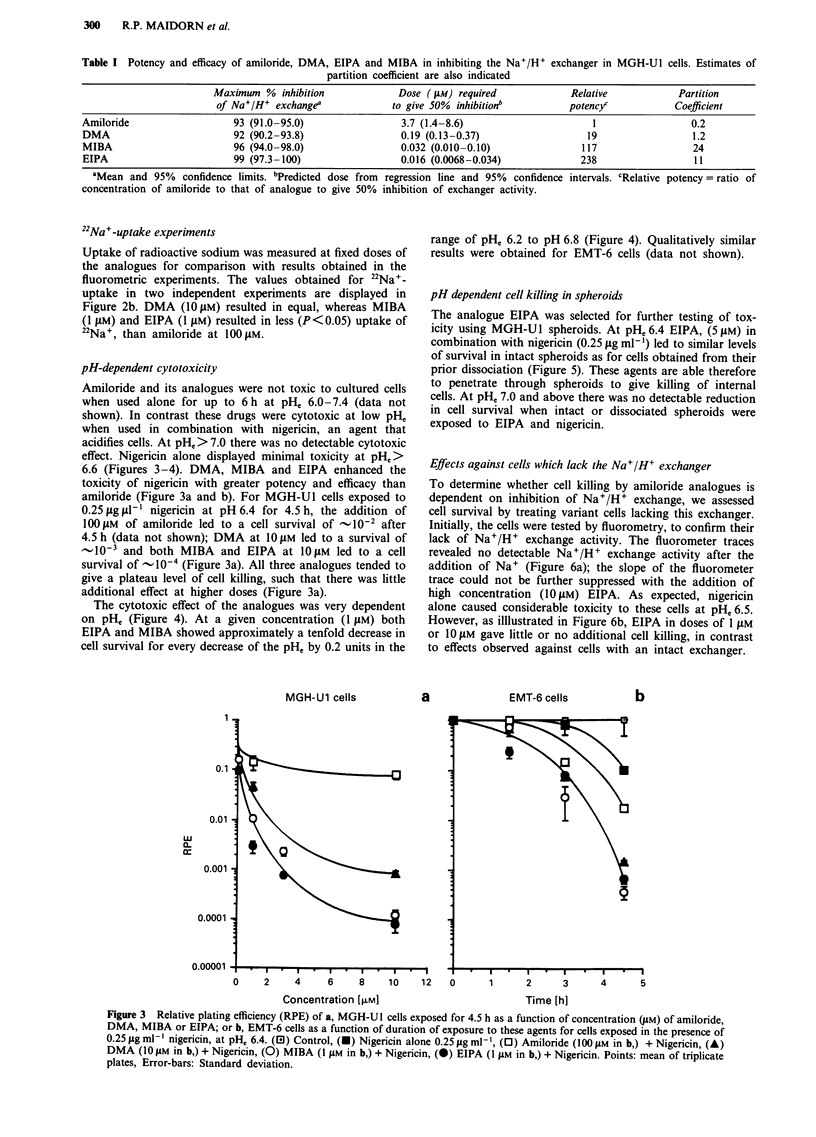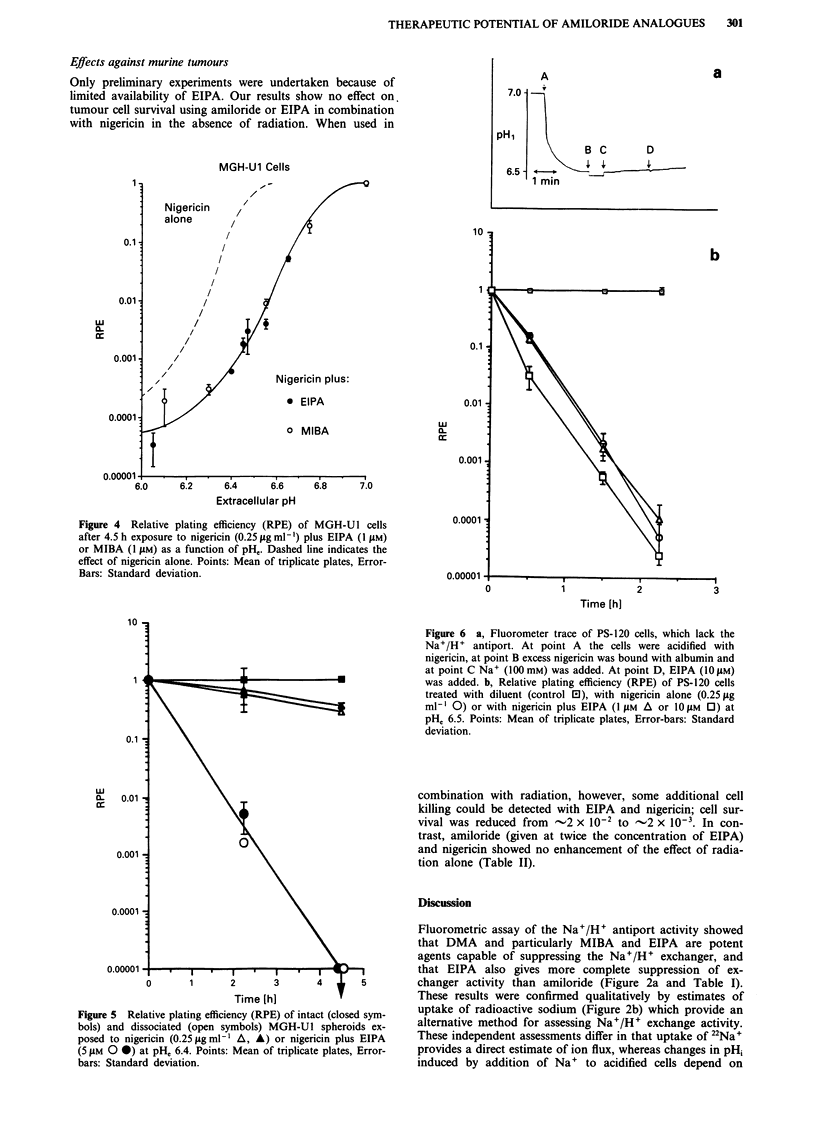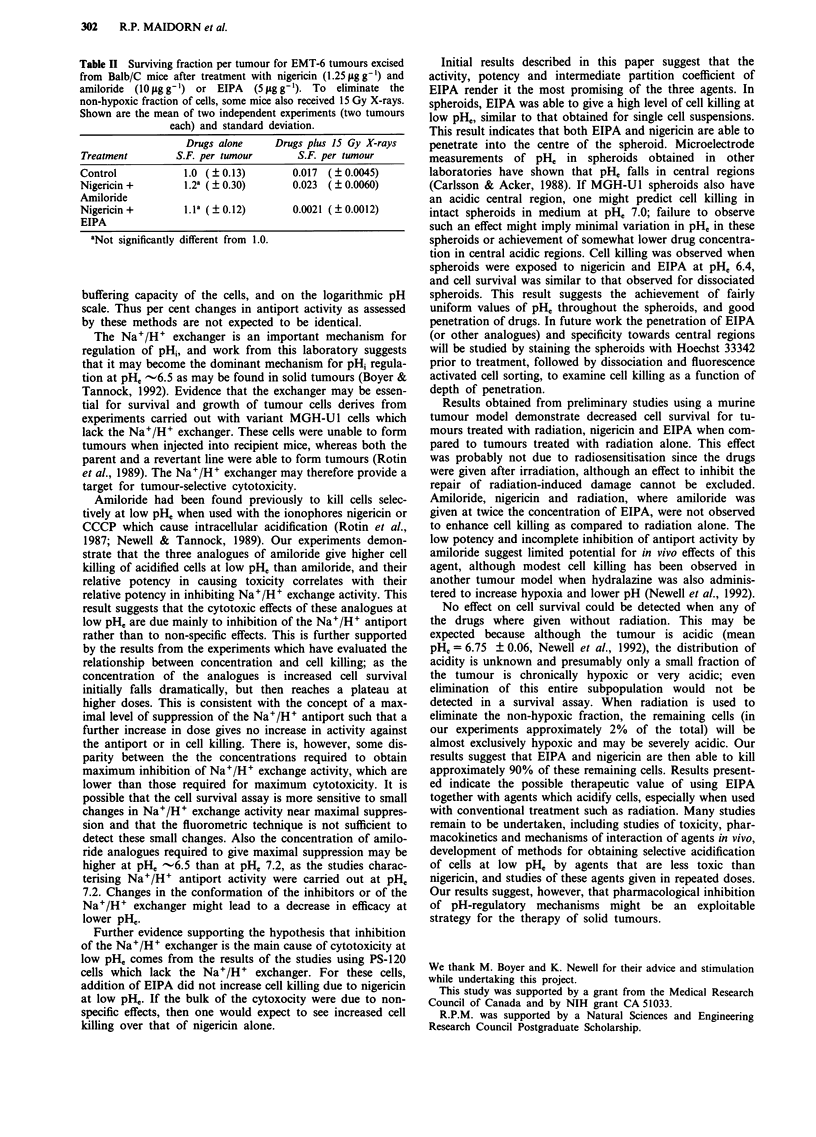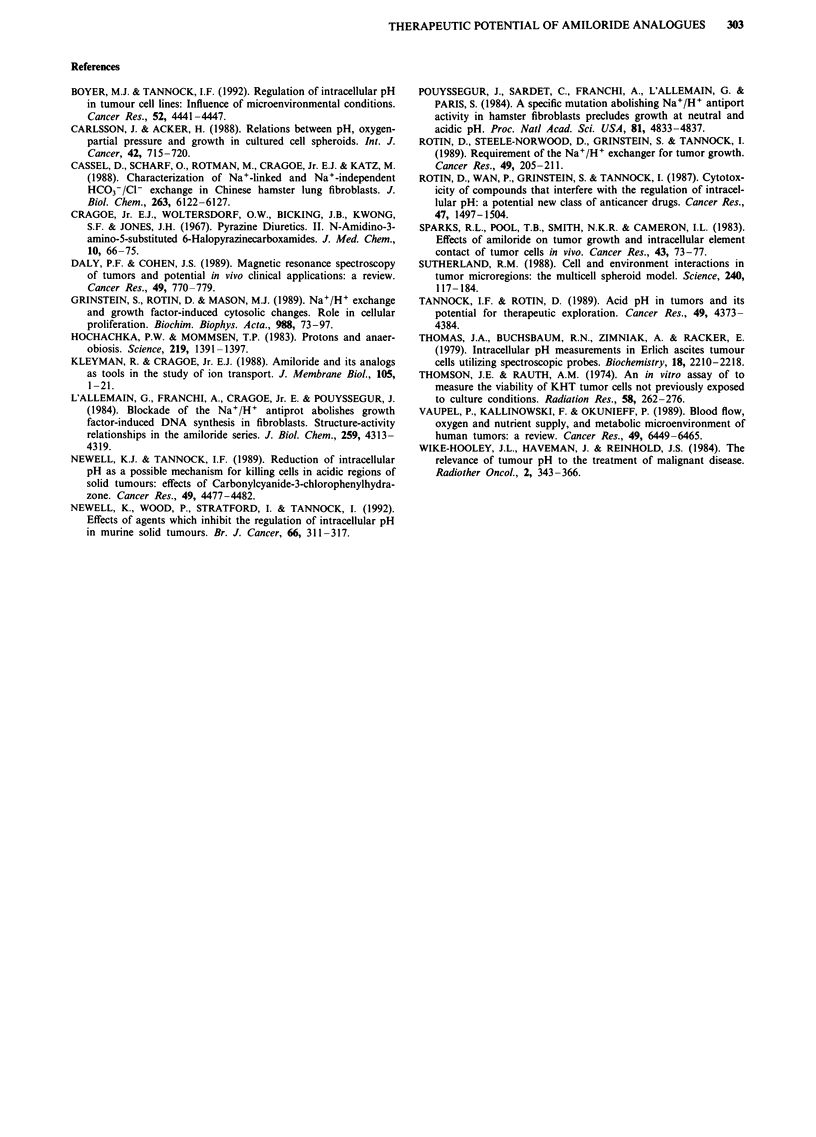# Therapeutic potential of analogues of amiloride: inhibition of the regulation of intracellular pH as a possible mechanism of tumour selective therapy.

**DOI:** 10.1038/bjc.1993.56

**Published:** 1993-02

**Authors:** R. P. Maidorn, E. J. Cragoe, I. F. Tannock

**Affiliations:** Department of Medical Biophysics, Ontario Cancer Institute, University of Toronto, Canada.

## Abstract

The extracellular pH (pHe) in solid tumours is frequently lower than the pHe in normal tissues. Cells within an acidic environment depend on mechanisms which regulate intracellular pH (pHi) for their survival, including the Na+/H+ antiport which exports protons in exchange for Na+ ions. Amiloride and its analogues DMA (5-(N,N-dimethyl)amiloride), MIBA (5-(N-methyl-N-isobutyl)amiloride) and EIPA (5-(N-ethyl-N-isopropyl)amiloride) are known to inhibit the Na+/H+ antiport and therefore decrease the cells ability to regulate pHi. All three analogues were found to be potent inhibitors of the antiport in human MGH-U1 and murine EMT-6 cells, with DMA being approximately 20, MIBA 100 and EIPA 200-fold as potent as amiloride; EIPA also gave more complete suppression of the Na+/H+ antiport. These agents were not toxic to cells when used alone; however, in combination with nigericin, an agent which acidifies cells, all three analogues were toxic to cells at pHe < 7.0, and markedly enhanced the toxicity of nigericin alone. Cell killing was greatest for nigericin used with EIPA or MIBA. None of the agents were toxic to cells at pHe 7.0 or above. When used against variant cells lacking the Na+/H+ antiport (PS-120 cells) EIPA did not enhance the cytotoxicity of nigericin alone, suggesting that the observed effect was due to inhibition of Na+/H+ exchange, rather than due to non-specific effects. The combination of EIPA and nigericin gave similar cell killing in previously dissociated and intact MGH-U1 spheroids, suggesting that the agents have good penetration of solid tissue. Preliminary experiments using EMT-6 tumours in mice suggested that EIPA and nigericin were able to enhance the toxicity of radiation in vivo, presumably through selective effects against the hypoxic (and probably acidic) subpopulation of cells that is resistant to radiation.


Br. J. Cancer (1993), 67, 297–303 © Macmillan Press Ltd., 1993

### Therapeutic potential of analogues of amiloride: inhibition of the regulation of intracellular pH as a possible mechanism of tumour selective therapy

R.P. Maidorn[1], E. J. Cragoe, Jr[2] & I.F. Tannock[1]

[1]*Department of Medical Biophysics, Ontario Cancer Institute, University of Toronto, 500 Sherbourne St., Toronto, Ontario, Canada, M4X 1K9;* [2]*PO Box 631548, Nacogdoches, Texas 75963-1548, USA.*

**Summary** The extracellular pH ($pH_e$) in solid tumours is frequently lower than the $pH_e$ in normal tissues. Cells within an acidic environment depend on mechanisms which regulate intracellular pH ($pH_i$) for their survival, including the $Na^+/H^+$ antiport which exports protons in exchange for $Na^+$ ions. Amiloride and its analogues DMA (5-(N,N-dimethyl)amiloride), MIBA (5-(N-methyl-N-isobutyl)amiloride) and EIPA (5-(N-ethyl-N-isopropyl)amiloride) are known to inhibit the $Na^+/H^+$ antiport and therefore decrease the cells ability to regulate $pH_i$. All three analogues were found to be potent inhibitors of the antiport in human MGH-U1 and murine EMT-6 cells, with DMA being approximately 20, MIBA 100 and EIPA 200-fold as potent as amiloride; EIPA also gave more complete suppression of the $Na^+/H^+$ antiport. These agents were not toxic to cells when used alone; however, in combination with nigericin, an agent which acidifies cells, all three analogues were toxic to cells at $pH_e < 7.0$, and markedly enhanced the toxicity of nigericin alone. Cell killing was greatest for nigericin used with EIPA or MIBA. None of the agents were toxic to cells at $pH_e$ 7.0 or above. When used against variant cells lacking the $Na^+/H^+$ antiport (PS-120 cells) EIPA did not enhance the cytotoxicity of nigericin alone, suggesting that the observed effect was due to inhibition of $Na^+/H^+$ exchange, rather than due to non-specific effects. The combination of EIPA and nigericin gave similar cell killing in previously dissociated and intact MGH-U1 spheroids, suggesting that the agents have good penetration of solid tissue. Preliminary experiments using EMT-6 tumours in mice suggested that EIPA and nigericin were able to enhance the toxicity of radiation *in vivo*, presumably through selective effects against the hypoxic (and probably acidic) subpopulation of cells that is resistant to radiation.

There are few consistent differences between properties of normal and malignant cells, and this has hindered the development of therapeutic agents that are selectively toxic to tumour cells. There are, however, important differences in the microenvironment of solid tumours and normal tissues. In a tumour the supporting vasculature is often not sufficient to provide a nutrient environment similar to that in normal tissues (Vaupel *et al.*, 1989), leading to regions within solid tumours that are not well perfused and which have a low influx of metabolites and a low efflux of potentially toxic catabolites. Due to the limited range of diffusion of oxygen within tissues, regions distal to blood vessels tend to become hypoxic. Solid tumours are also known to be more acidic than normal tissues (Wike-Hooley *et al.*, 1984). Hypoxic regions of tumours are likely to be particularly acidic, because of enforced dependence on anaerobic glycolysis as a major source of metabolic energy. The net production of protons from the formation of lactic acid and the hydrolysis of ATP is though to lead to a decrease in pH (Hochachka & Mommsen, 1983). Measurements of extracellular tumour pH ($pH_e$), mainly by insertion of micro-electrodes, have confirmed that tumours are on average 0.5 pH units lower than normal tissue, with tumour $pH_e$ usually in the range from pH 6.5 to pH 7.0 and normal tissue $pH_e$ between pH 7.1 to pH 7.6 (Wike-Hooley *et al.*, 1984). Measurements by $^{31}$P-NMR-spectroscopy, which indicate mainly intracellular pH ($pH_i$), have shown no significant differences between $pH_i$ in solid tumours as compared to normal tissue, and in brain tumours slightly elevated levels of $pH_i$ have been recorded (Daly & Cohen, 1989; Vaupel *et al.*, 1989). The difference between measurements of $pH_i$ and $pH_e$ may be explained by the presence of mechanisms which regulate $pH_i$ in the face of an acid load.

Correspondence: I.F. Tannock.
Received 21 July 1992.

The difference in $pH_e$ between tumour and normal tissue provides an opportunity for tumour-selective therapy through the development of drugs whose toxicity is greater towards cells at lower $pH_e$ (Tannock & Rotin, 1989). One possible approach to such therapy would be the development of agents that are able to inhibit the mechanisms which regulate $pH_i$, therefore leading to intracellular acidification in the presence of an acidic environment and death of those cells which are at low $pH_e$. Cells in an acidic environment depend on the presence of membrane based ion transport systems to maintain their $pH_i$ within the normal range ($pH_i$ 7.2). The two major exchangers known to be involved with pH regulation under acidic conditions are the stilbene-sensitive $Na^+$ dependent $HCO_3^-/Cl^-$ exchanger (Cassel *et al.*, 1988) and the amiloride-sensitive $Na^+/H^+$ antiport (Grinstein *et al.*, 1989). The latter has been found in most animal cells and exchanges extracellular $Na^+$ with intracellular $H^+$ with a 1:1 stoichiometry. The inward $Na^+$ gradient, which is maintained by the sodium-potassium-ATPase, drives the exchanger. When the cells produce protons the exchanger can protect the cell by exporting $H^+$ in exchange for $Na^+$, thus minimising or preventing any decrease in $pH_i$. The importance of the $Na^+/H^+$ exchanger for normal tumour growth has been suggested by the inability of variant MGH-U1 (human bladder cancer) cells lacking the $Na^+/H^+$ exchanger to form tumours in immune deficient mice (Rotin *et al.*, 1989).

Amiloride and some of its analogues are able to inhibit the $Na^+/H^+$ antiport (Cragoe *et al.*, 1967; L'Allemain *et al.*, 1984; Kleyman & Cragoe, 1988). In previous experiments amiloride significantly enhanced the pH-dependent cytotoxic effect of the ionophores nigericin and CCCP (Rotin *et al.*, 1987; Newell & Tannock, 1989). Sparks *et al.* (1983) reported suppression of growth of DMA/J mammary carcinoma and H6 hepatoma in mice during repeated treatments with amiloride. Substitution at the 5-amino group of amiloride with certain lipophilic substituents has generated analogues that have been reported to have much greater potency for inhibiting the $Na^+/H^+$ antiport than the parent compound (Cragoe *et al.*, 1967; L'Allemain *et al.*, 1984; Kleyman & Cragoe, 1988). We herein describe studies of three amiloride analogues: DMA, MIBA and EIPA, which were shown previously to be more potent inhibitors of the $Na^+/H^+$ antiport than amiloride. We have studied these compounds by quantitating their ability to suppress the $Na^+/H^+$ antiport in tumour cells and their efficiency in causing $pH_e$ dependent cell killing when used alone or with agents that acidify cells at low $pH_e$. The analogues were tested for toxicity against single cell suspensions and against spheroids, and preliminary *in vivo* experiments were undertaken using a murine tumour model.

### Materials and methods

### *Cells*

The following cell lines were used in these experiments: MGH-U1 cells were derived from a human bladder carcinoma, (obtained from Dr G. Prout, Massachusetts General Hospital, Boston, MA), EMT-6 a mouse mammary sarcoma line, (obtained from Dr R. Sutherland, Rochester, NY), and PS-120 lung fibroblast cells, a variant Chinese hamster line lacking the $Na^+/H^+$ exchanger, (obtained from Dr J. Pouyssegur, Université de Nice, France), (Pouyssegur *et al.*, 1984). Cell lines were maintained in α-MEM containing 5% FCS and kanamycin using standard culture techniques. Cultures were reestablished from frozen stock after approximately 20 passages, and were tested periodically to ensure absence of mycoplasma. All experiments were performed using exponentially growing cells.

### *Chemicals*

DMA, MIBA and EIPA were synthesised by one of us at Merck Sharpe and Dohme, New Jersey. For stock solutions amiloride was dissolved in double de-ionised $H_2O$; DMA, MIBA and EIPA were dissolved in 2% DMSO and then brought to the final concentration with 4 × distilled $H_2O$. BCECF-AM was purchased from Molecular Probes (Eugene, OR). Nigericin, amiloride and all other chemicals were purchased from Sigma (St. Louis, MO).

### *Quantitation of $Na^+/H^+$ exchanger activity*

Exponentially growing cells were detached from their flasks using 0.025% trypsin and 0.01% EDTA, washed and resuspended in α-MEM without FCS at a final concentration of $1.5 \times 10^6$ cells $ml^{-1}$ in 2 ml. The cells were then incubated for 30 mins with 2 $\mu g\ ml^{-1}$ of the tetraacetoxymethyl ester of BCECF (BCECF-AM). BCECF-AM is uncharged and therefore able to diffuse across the cell membrane. Once inside the cell BCECF-AM is cleaved by non-specific esterases, producing the charged, highly fluorescent and poorly permeable BCECF, which remains trapped within the cell. After incubation in BCECF-AM, 320 µl of the suspension was removed, centrifuged and resuspended in 80 µl of α-MEM and placed in a cuvet containing 1.8 ml of sodium- and bicarbonate free NMG-buffer (140 mM NMG, 10 mM Glucose, 1 mM KCl, 1 mM $CaCl_2$, 1 mM $MgCl_2$, pH 7.2). Measurements of $pH_i$ were made using a Perkin Elmer LS3 fluorescence spectrophotometer, with excitation and emission wavelengths set to 495 nm and 525 nm respectively. At these wavelengths, the fluorescence of BCECF is linearly related to $pH_i$ in the range of $pH_i$ 6.0 to 7.5 At an excitation wavelength of 440 nm, fluorescence emission (at 525 nm) of BCECF is independent of pH, and this was used as a check for dye leakage from cells. In our experiments such leakage was minimal, and it was not therefore necessary to use a ratio of fluorescence intensities to estimate $pH_i$.

To determine the activity of the $Na^+/H^+$ antiport, cells were first acidified to $pH_i$ 6.5 using a fixed concentration of nigericin, an ionophore which allows extracellular protons to exchange across the cell membrane for intracellular potassium (Thomas *et al.*, 1979). Excess nigericin was bound with albumin. Activity of the $Na^+/H^+$ antiport in the presence or absence of an inhibitor was then quantitated by adding NaCl to the cuvette. The addition of NaCl (to a final concentration of 100 mM) allows the cells to use their $Na^+/H^+$ antiport to raise $pH_i$ and the rate of increase in $pH_i$ is a direct measure of the activity of the $Na^+/H^+$ exchanger. In the presence of amiloride or its analogues the rise in $pH_i$ is inhibited; the per cent inhibition is measured by the ratio of the slopes of the fluorometer lines ($\Delta pH_i/\Delta t$) after adding $Na^+$, and expressed as a function of concentration of the inhibitor (Figure 1). Although the addition of NaCl causes an increase in osmolality, control experiments in which a similar rise in osmolality was caused by adding NMG or $K^+$ containing media did not activate the $Na^+/H^+$ exchanger.

### *$^{22}Na^+$ uptake*

The uptake of $^{22}Na^+$ into cells provides an independent method for assessment of $Na^+/H^+$ exchange activity (Pouyssegur *et al.*, 1984). Cells were seeded in multiwell trays and grown for 2 days in α-MEM + 5% FCS. At this point the media was replaced with $NH_4Cl$-containing media (α-MEM without $NaHCO_3$ plus 50 mM $NH_4Cl$, 0.1 mM uridine, 0.1 mM hypoxanthine, and 20 mm Hepes, pH 7.4) and cells were incubated for 30 min at 37°C. Transient exposure to $NH_4Cl$ is able to acidify cells (Pouyssegur *et al.*, 1984). Cells were then rinsed with 140 mM $NMG^+Cl^-$ (pH 7.4) and incubated in $^{22}Na^+$ solution (135 mM $NMG^+Cl^-$, 1 mM $MgCl_2$, 2 mM $CaCl_2$, 1 mM NaCl, 2.5 $\mu Ci\ ml^{-1}$ $^{22}NaCl$, 1 mM ouabain, 20 mM HEPES-Tris, pH 7.4) for 6 min. The presence of ouabain inhibits the $Na^+/K^+$ ATPase, and prevents $Na^+$-transport by this mechanism. To some multiwells a given amount of amiloride or the analogue was added prior to the addition of $^{22}Na^+$. The multiwells were then rinsed three times with ice-cold phosphate-buffered saline. Cells were disrupted with Triton X-100, and uptake of $^{22}Na^+$ was measured using a scintillation counter.

### *Partition coefficient*

The partition coefficient is a measure of the ratio of lipid to water solubility of a given compound. The partition coefficients for amiloride and the analogues were determined by dissolving known amounts of the compounds in a 1:1 water:octanol emulsion by vigorous shaking for 10–15 min. The two phases were then allowed to separate and the concentration of amiloride or its analogues was obtained by measuring the absorbance of each phase using a spectrophotometer.

**Figure 1** Schematic fluorometer trace of cells loaded with BCECF and suspended in sodium-free NMG-buffer. At point **A** cells are acidified with nigericin, at point **B** albumin is added to bind excess nigericin. At point C NaCl is added to allow regulation of $pH_i$. In the presence of an inhibitor of the $Na^+/H^+$ antiport (e.g. amiloride) the rate of recovery of $pH_i$ decreases. Per cent activity of the antiport is expressed as the ratio of slopes of the traces (after adding NaCl) in the presence and absence of the inhibitor.

### *Cell survival experiments*

Cell survival was assessed by measuring plating efficiency after exposure to compounds at different levels of $pH_e$. Exponentially growing cells were detached from their flasks using 0.025% trypsin and 0.01% EDTA, washed and resuspended in pH balanced α-MEM and 5% FCS at a final concentration of $10^6$ cells $ml^{-1}$. The $pH_e$ of the medium was buffered to the desired value in the range of 6.0–7.4 by adding appropriate amounts of α-MEM + bicarbonate (25 mM) + 5% FCS to α-MEM + Hepes (25 mM) + 5% FCS. Aliquots of 5 ml were transferred to small glass vials and stirred at 37°C. Humidified gas containing 5% $CO_2$ and air balance was passed through the vials. After 30 min of incubation, the compounds were added in a volume of 125–250 μl; control vials received equivalent amounts of diluent. At given time intervals, 0.5 ml samples were removed by syringe. The cells were centrifuged, resuspended in fresh αMEM + 5% FCS and counted. Serial dilutions of the cells were then plated in triplicate in α-MEM + 5% FCS in petri dishes. After an incubation time of 9–13 days, colonies were stained with methylene blue and counted. Buffering of $pH_e$ in the culture media achieved control of + / − 0.15 pH units. All experiments were repeated but the critical dependance of results on $pH_e$ leads to some variation among replicate experiments. Results of single experiments are shown for illustration, but replicate experiments always gave qualitatively similar results.

### *Experiments with spheroids*

Spheroids provide a model of intermediate complexity between tissue culture and tumours in experimental animals, that allows for cell-cell interaction and tissue penetration of toxic agents (Sutherland, 1988). We have therefore studied the toxicity of nigericin and analogues of amiloride towards cells in MGH-U1 spheroids. The spheroids were grown in 200 ml spinner flasks for 12–14 days, by which time the spheroids had an average diameter of 800 μm. The spheroids were then washed in phosphate-buffered saline and resuspended in 50 ml of pH-balanced media. Intact spheroids or cells from dissociated spheroids were exposed to agents at defined levels of $pH_e$. Spheroids were dissociated in 0.025% trypsin and 0.01% EDTA and the cells were centrifuged and resuspended in 50 ml of pH-balanced media. Spheroids or single cells were exposed in spinner flasks in the presence of medium that was buffered to the desired $pH_e$ as described above. Spinner flasks were gassed with 5% $CO_2$ to stabilise the $pH_e$ of the medium. After 30 min of incubation the agents were added (time 0). Control groups received an equivalent amount of diluent. Samples were taken at given time intervals and intact spheroids were then dissociated using 0.025% trypsin and 0.01% EDTA. All samples (intact and dissociated) were then centrifuged and resuspended in fresh α-MEM media + 5% FCS, counted, and plated in serial dilution. Clonogenic survival was determined after 9–11 days, as described above.

### *Experiments using murine tumours*

Because of limited availability of amiloride analogues, only preliminary *in vivo* experiments were performed, using a murine tumour model. The left hind legs of Balb/c-mice were injected with syngeneic EMT-6 cells. Treatment of the mice began when the tumour-bearing leg had reached a diameter of 8.5–9.5 mm (equivalent to tumour weight of 0.3–0.5 g), usually 6–7 days after injection. The mice were then injected intraperitoneally with either amiloride (10 μg $g^{-1}$) or EIPA (5 μg $g^{-1}$), in combination with 1.25 μg $g^{-1}$ nigericin (these doses were tolerated by the animals without visible effects. When the doses of EIPA and nigericin were increased to 10 μg $g^{-1}$ and 2.5 μg $g^{-1}$ approximately 50% of the animals died within 24 h). Microelectrode measurements performed in this laboratory have shown that EMT-6 tumours develop an acidic microenvironment with a mean $pH_e$ of 6.75 + / − 0.06 (Newell *et al.*, 1992). To kill the aerobic (and probably less acidic) subpopulation of the tumours, the tumour bearing left hind legs of some mice were also irradiated with 15 Gray X-rays, and drugs were given within 15 min after radiation. After a period of 18–24 h the tumours were excised, weighed and dissociated by passing them through a coarse screen followed by incubation in trypsin and DNase I for 30 min as described previously (Thomson & Rauth, 1974). The cells were then centrifuged and resuspended in 8 ml of α-MEM + 5% FCS, passed through a final screen and counted. Serial dilutions were plated and clonogenic survival was assayed, as described above.

### Results

### *Inhibition of the $Na^+/H^+$ antiport assessed by fluorometry*

The results of multiple experiments which have characterised the dose response relationship of amiloride and its analogues for inhibition of $Na^+/H^+$ exchange activity in MGH-U1 cells are summarised in Figure 2a and Table I. Similar results were found for EMT-6 cells. Also shown in Table I are the partition coefficients determined for each analogue. Each of the analogues is more potent than amiloride, and EIPA and MIBA are the most potent and complete inhibitors of $Na^+/H^+$ exchanger activity.

**Figure 2** **a**, Per cent activity of the $Na^+/H^+$ exchanger in MGH-U1 cells in the presence of different concentrations of amiloride, DMA, MIBA or EIPA. Per cent activity was measured as the rate of recovery of $pH_i$ relative to control (no inhibitor) after intracellular acidification with nigericin and addition of $Na^+$. Points: Mean of a minimum of two experiments, Error-bars: Standard deviation. **b**, Per cent uptake of $^{22}Na^+$ by MGH-U1 cells over 6 min after acidification with $NH_4Cl$ media. Columns: Mean of eight measurements from two individual experiments, Error-bars: 95% confidence intervals.

**Table I** Potency and efficacy of amiloride, DMA, EIPA and MIBA in inhibiting the $Na^+/H^+$ exchanger in MGH-U1 cells. Estimates of partition coefficient are also indicated

| | *Maximum % inhibition of $Na^+/H^+$ exchange*[a] | *Dose (μM) required to give 50% inhibition*[b] | *Relative potency*[c] | *Partition Coefficient* |
|---|---|---|---|---|
| Amiloride | 93 (91.0–95.0) | 3.7 (1.4–8.6) | 1 | 0.2 |
| DMA | 92 (90.2–93.8) | 0.19 (0.13–0.37) | 19 | 1.2 |
| MIBA | 96 (94.0–98.0) | 0.032 (0.010–0.10) | 117 | 24 |
| EIPA | 99 (97.3–100) | 0.016 (0.0068–0.034) | 238 | 11 |

[a]Mean and 95% confidence limits. [b]Predicted dose from regression line and 95% confidence intervals. [c]Relative potency = ratio of concentration of amiloride to that of analogue to give 50% inhibition of exchanger activity.

### *$^{22}Na^+$-uptake experiments*

Uptake of radioactive sodium was measured at fixed doses of the analogues for comparison with results obtained in the fluorometric experiments. The values obtained for $^{22}Na^+$-uptake in two independent experiments are displayed in Figure 2b. DMA (10 μM) resulted in equal, whereas MIBA (1 μM) and EIPA (1 μM) resulted in less ($P < 0.05$) uptake of $^{22}Na^+$, than amiloride at 100 μM.

### *pH-dependent cytotoxicity*

Amiloride and its analogues were not toxic to cultured cells when used alone for up to 6 h at $pH_e$ 6.0–7.4 (data not shown). In contrast these drugs were cytotoxic at low $pH_e$ when used in combination with nigericin, an agent that acidifies cells. At $pH_e > 7.0$ there was no detectable cytotoxic effect. Nigericin alone displayed minimal toxicity at $pH_e >$ 6.6 (Figures 3–4). DMA, MIBA and EIPA enhanced the toxicity of nigericin with greater potency and efficacy than amiloride (Figure 3a and b). For MGH-U1 cells exposed to 0.25 μg $\mu l^{-1}$ nigericin at pH 6.4 for 4.5 h, the addition of 100 μM of amiloride led to a cell survival of $\sim 10^{-2}$ after 4.5 h (data not shown); DMA at 10 μM led to a survival of $\sim 10^{-3}$ and both MIBA and EIPA at 10 μM led to a cell survival of $\sim 10^{-4}$ (Figure 3a). All three analogues tended to give a plateau level of cell killing, such that there was little additional effect at higher doses (Figure 3a).

The cytotoxic effect of the analogues was very dependent on $pH_e$ (Figure 4). At a given concentration (1 μM) both EIPA and MIBA showed approximately a tenfold decrease in cell survival for every decrease of the $pH_e$ by 0.2 units in the range of $pH_e$ 6.2 to pH 6.8 (Figure 4). Qualitatively similar results were obtained for EMT-6 cells (data not shown).

### *pH dependent cell killing in spheroids*

The analogue EIPA was selected for further testing of toxicity using MGH-U1 spheroids. At $pH_e$ 6.4 EIPA, (5 μM) in combination with nigericin (0.25 μg $ml^{-1}$) led to similar levels of survival in intact spheroids as for cells obtained from their prior dissociation (Figure 5). These agents are able therefore to penetrate through spheroids to give killing of internal cells. At $pH_e$ 7.0 and above there was no detectable reduction in cell survival when intact or dissociated spheroids were exposed to EIPA and nigericin.

### *Effects against cells which lack the $Na^+/H^+$ exchanger*

To determine whether cell killing by amiloride analogues is dependent on inhibition of $Na^+/H^+$ exchange, we assessed cell survival by treating variant cells lacking this exchanger. Initially, the cells were tested by fluorometry, to confirm their lack of $Na^+/H^+$ exchange activity. The fluorometer traces revealed no detectable $Na^+/H^+$ exchange activity after the addition of $Na^+$ (Figure 6a); the slope of the fluorometer trace could not be further suppressed with the addition of high concentration (10 μM) EIPA. As expected, nigericin alone caused considerable toxicity to these cells at $pH_e$ 6.5. However, as illlustrated in Figure 6b, EIPA in doses of 1 μM or 10 μM gave little or no additional cell killing, in contrast to effects observed against cells with an intact exchanger.

**Figure 3** Relative plating efficiency (RPE) of **a**, MGH-U1 cells exposed for 4.5 h as a function of concentration (μM) of amiloride, DMA, MIBA or EIPA; or **b**, EMT-6 cells as a function of duration of exposure to these agents for cells exposed in the presence of 0.25 μg $ml^{-1}$ nigericin, at $pH_e$ 6.4. (⊡) Control, (■) Nigericin alone 0.25 μg $ml^{-1}$, (□) Amiloride (100 μM in **b**,) + Nigericin, (▲) DMA (10 μM in **b**,) + Nigericin, (○) MIBA (1 μM in **b**,) + Nigericin, (●) EIPA (1 μM in **b**,) + Nigericin. Points: mean of triplicate plates, Error-bars: Standard deviation.

*Effects against murine tumours*

Only preliminary experiments were undertaken because of limited availability of EIPA. Our results show no effect on tumour cell survival using amiloride or EIPA in combination with nigericin in the absence of radiation. When used in combination with radiation, however, some additional cell killing could be detected with EIPA and nigericin; cell survival was reduced from ~$2 \times 10^{-2}$ to ~$2 \times 10^{-3}$. In contrast, amiloride (given at twice the concentration of EIPA) and nigericin showed no enhancement of the effect of radiation alone (Table II).

**Figure 4** Relative plating efficiency (RPE) of MGH-U1 cells after 4.5 h exposure to nigericin (0.25 μg $ml^{-1}$) plus EIPA (1 μM) or MIBA (1 μM) as a function of $pH_e$. Dashed line indicates the effect of nigericin alone. Points: Mean of triplicate plates, Error-Bars: Standard deviation.

**Figure 5** Relative plating efficiency (RPE) of intact (closed symbols) and dissociated (open symbols) MGH-U1 spheroids exposed to nigericin (0.25 μg $ml^{-1}$ Δ, ▲) or nigericin plus EIPA (5 μM ○ ●) at $pH_e$ 6.4. Points: Mean of triplicate plates, Error-bars: Standard deviation.

**Figure 6** **a**, Fluorometer trace of PS-120 cells, which lack the $Na^+/H^+$ antiport. At point A the cells were acidified with nigericin, at point B excess nigericin was bound with albumin and at point C $Na^+$ (100 mM) was added. At point D, EIPA (10 μM) was added. **b**, Relative plating efficiency (RPE) of PS-120 cells treated with diluent (control ⊡), with nigericin alone (0.25 μg $ml^{-1}$ ○) or with nigericin plus EIPA (1 μM Δ or 10 μM □) at $pH_e$ 6.5. Points: Mean of triplicate plates, Error-bars: Standard deviation.

### Discussion

Fluorometric assay of the $Na^+/H^+$ antiport activity showed that DMA and particularly MIBA and EIPA are potent agents capable of suppressing the $Na^+/H^+$ exchanger, and that EIPA also gives more complete suppression of exchanger activity than amiloride (Figure 2a and Table I). These results were confirmed qualitatively by estimates of uptake of radioactive sodium (Figure 2b) which provide an alternative method for assessing $Na^+/H^+$ exchange activity. These independent assessments differ in that uptake of $^{22}Na^+$ provides a direct estimate of ion flux, whereas changes in $pH_i$ induced by addition of $Na^+$ to acidified cells depend on

**Table II** Surviving fraction per tumour for EMT-6 tumours excised from Balb/C mice after treatment with nigericin ($1.25\,\mu g\,g^{-1}$) and amiloride ($10\,\mu g\,g^{-1}$) or EIPA ($5\,\mu g\,g^{-1}$). To eliminate the non-hypoxic fraction of cells, some mice also received 15 Gy X-rays. Shown are the mean of two independent experiments (two tumours each) and standard deviation.

| *Treatment* | *Drugs alone S.F. per tumour* | *Drugs plus 15 Gy X-rays S.F. per tumour* |
|---|---|---|
| Control | 1.0 (± 0.13) | 0.017 (± 0.0045) |
| Nigericin + Amiloride | 1.2[a] (± 0.30) | 0.023 (± 0.0060) |
| Nigericin + EIPA | 1.1[a] (± 0.12) | 0.0021 (± 0.0012) |

[a]Not significantly different from 1.0.

buffering capacity of the cells, and on the logarithmic pH scale. Thus per cent changes in antiport activity as assessed by these methods are not expected to be identical.

The $Na^+/H^+$ exchanger is an important mechanism for regulation of $pH_i$, and work from this laboratory suggests that it may become the dominant mechanism for $pH_i$ regulation at $pH_e$ ~6.5 as may be found in solid tumours (Boyer & Tannock, 1992). Evidence that the exchanger may be essential for survival and growth of tumour cells derives from experiments carried out with variant MGH-U1 cells which lack the $Na^+/H^+$ exchanger. These cells were unable to form tumours when injected into recipient mice, whereas both the parent and a revertant line were able to form tumours (Rotin *et al.*, 1989). The $Na^+/H^+$ exchanger may therefore provide a target for tumour-selective cytotoxicity.

Amiloride had been found previously to kill cells selectively at low $pH_e$ when used with the ionophores nigericin or CCCP which cause intracellular acidification (Rotin *et al.*, 1987; Newell & Tannock, 1989). Our experiments demonstrate that the three analogues of amiloride give higher cell killing of acidified cells at low $pH_e$ than amiloride, and their relative potency in causing toxicity correlates with their relative potency in inhibiting $Na^+/H^+$ exchange activity. This result suggests that the cytotoxic effects of these analogues at low $pH_e$ are due mainly to inhibition of the $Na^+/H^+$ antiport rather than to non-specific effects. This is further supported by the results from the experiments which have evaluated the relationship between concentration and cell killing; as the concentration of the analogues is increased cell survival initially falls dramatically, but then reaches a plateau at higher doses. This is consistent with the concept of a maximal level of suppression of the $Na^+/H^+$ antiport such that a further increase in dose gives no increase in activity against the antiport or in cell killing. There is, however, some disparity between the the concentrations required to obtain maximum inhibition of $Na^+/H^+$ exchange activity, which are lower than those required for maximum cytotoxicity. It is possible that the cell survival assay is more sensitive to small changes in $Na^+/H^+$ exchange activity near maximal suppression and that the fluorometric technique is not sufficient to detect these small changes. Also the concentration of amiloride analogues required to give maximal suppression may be higher at $pH_e$ ~6.5 than at $pH_e$ 7.2, as the studies characterising $Na^+/H^+$ antiport activity were carried out at $pH_e$ 7.2. Changes in the conformation of the inhibitors or of the $Na^+/H^+$ exchanger might lead to a decrease in efficacy at lower $pH_e$.

Further evidence supporting the hypothesis that inhibition of the $Na^+/H^+$ exchanger is the main cause of cytotoxicity at low $pH_e$ comes from the results of the studies using PS-120 cells which lack the $Na^+/H^+$ exchanger. For these cells, addition of EIPA did not increase cell killing due to nigericin at low $pH_e$. If the bulk of the cytoxocity were due to non-specific effects, then one would expect to see increased cell killing over that of nigericin alone.

Initial results described in this paper suggest that the activity, potency and intermediate partition coefficient of EIPA render it the most promising of the three agents. In spheroids, EIPA was able to give a high level of cell killing at low $pH_e$, similar to that obtained for single cell suspensions. This result indicates that both EIPA and nigericin are able to penetrate into the centre of the spheroid. Microelectrode measurements of $pH_e$ in spheroids obtained in other laboratories have shown that $pH_e$ falls in central regions (Carlsson & Acker, 1988). If MGH-U1 spheroids also have an acidic central region, one might predict cell killing in intact spheroids in medium at $pH_e$ 7.0; failure to observe such an effect might imply minimal variation in $pH_e$ in these spheroids or achievement of somewhat lower drug concentration in central acidic regions. Cell killing was observed when spheroids were exposed to nigericin and EIPA at $pH_e$ 6.4, and cell survival was similar to that observed for dissociated spheroids. This result suggests the achievement of fairly uniform values of $pH_e$ throughout the spheroids, and good penetration of drugs. In future work the penetration of EIPA (or other analogues) and specificity towards central regions will be studied by staining the spheroids with Hoechst 33342 prior to treatment, followed by dissociation and fluorescence activated cell sorting, to examine cell killing as a function of depth of penetration.

Results obtained from preliminary studies using a murine tumour model demonstrate decreased cell survival for tumours treated with radiation, nigericin and EIPA when compared to tumours treated with radiation alone. This effect was probably not due to radiosensitisation since the drugs were given after irradiation, although an effect to inhibit the repair of radiation-induced damage cannot be excluded. Amiloride, nigericin and radiation, where amiloride was given at twice the concentration of EIPA, were not observed to enhance cell killing as compared to radiation alone. The low potency and incomplete inhibition of antiport activity by amiloride suggest limited potential for *in vivo* effects of this agent, although modest cell killing has been observed in another tumour model when hydralazine was also administered to increase hypoxia and lower pH (Newell *et al.*, 1992).

No effect on cell survival could be detected when any of the drugs where given without radiation. This may be expected because although the tumour is acidic (mean $pH_e = 6.75 \pm 0.06$, Newell *et al.*, 1992), the distribution of acidity is unknown and presumably only a small fraction of the tumour is chronically hypoxic or very acidic; even elimination of this entire subpopulation would not be detected in a survival assay. When radiation is used to eliminate the non-hypoxic fraction, the remaining cells (in our experiments approximately 2% of the total) will be almost exclusively hypoxic and may be severely acidic. Our results suggest that EIPA and nigericin are then able to kill approximately 90% of these remaining cells. Results presented indicate the possible therapeutic value of using EIPA together with agents which acidify cells, especially when used with conventional treatment such as radiation. Many studies remain to be undertaken, including studies of toxicity, pharmacokinetics and mechanisms of interaction of agents *in vivo*, development of methods for obtaining selective acidification of cells at low $pH_e$ by agents that are less toxic than nigericin, and studies of these agents given in repeated doses. Our results suggest, however, that pharmacological inhibition of pH-regulatory mechanisms might be an exploitable strategy for the therapy of solid tumours.

We thank M. Boyer and K. Newell for their advice and stimulation while undertaking this project.

This study was supported by a grant from the Medical Research Council of Canada and by NIH grant CA 51033.

R.P.M. was supported by a Natural Sciences and Engineering Research Council Postgraduate Scholarship.

### References

BOYER, M.J. & TANNOCK, I.F. (1992). Regulation of intracellular pH in tumour cell lines: Influence of microenvironmental conditions. *Cancer Res.*, **52**, 4441–4447.

CARLSSON, J. & ACKER, H. (1988). Relations between pH, oxygen-partial pressure and growth in cultured cell spheroids. *Int. J. Cancer*, **42**, 715–720.

CASSEL, D., SCHARF, O., ROTMAN, M., CRAGOE, Jr. E.J. & KATZ, M. (1988). Characterization of $Na^+$-linked and $Na^+$-independent $HCO_3^-/Cl^-$ exchange in Chinese hamster lung fibroblasts. *J. Biol. Chem.*, **263**, 6122–6127.

CRAGOE, Jr. E.J., WOLTERSDORF, O.W., BICKING, J.B., KWONG, S.F. & JONES, J.H. (1967). Pyrazine Diuretics. II. N-Amidino-3-amino-5-substituted 6-Halopyrazinecarboxamides. *J. Med. Chem.*, **10**, 66–75.

DALY, P.F. & COHEN, J.S. (1989). Magnetic resonance spectroscopy of tumors and potential *in vivo* clinical applications: a review. *Cancer Res.*, **49**, 770–779.

GRINSTEIN, S., ROTIN, D. & MASON, M.J. (1989). $Na^+/H^+$ exchange and growth factor-induced cytosolic changes. Role in cellular proliferation. *Biochim. Biophys. Acta.*, **988**, 73–97.

HOCHACHKA, P.W. & MOMMSEN, T.P. (1983). Protons and anaerobiosis. *Science*, **219**, 1391–1397.

KLEYMAN, R. & CRAGOE, Jr. E.J. (1988). Amiloride and its analogs as tools in the study of ion transport. *J. Membrane Biol.*, **105**, 1–21.

L'ALLEMAIN, G., FRANCHI, A., CRAGOE, Jr. E. & POUYSSEGUR, J. (1984). Blockade of the $Na^+/H^+$ antiprot abolishes growth factor-induced DNA synthesis in fibroblasts. Structure-activity relationships in the amiloride series. *J. Biol. Chem.*, **259**, 4313–4319.

NEWELL, K.J. & TANNOCK, I.F. (1989). Reduction of intracellular pH as a possible mechanism for killing cells in acidic regions of solid tumours: effects of Carbonylcyanide-3-chlorophenylhydrazone. *Cancer Res.*, **49**, 4477–4482.

NEWELL, K., WOOD, P., STRATFORD, I. & TANNOCK, I. (1992). Effects of agents which inhibit the regulation of intracellular pH in murine solid tumours. *Br. J. Cancer*, **66**, 311–317.

POUYSSEGUR, J., SARDET, C., FRANCHI, A., L'ALLEMAIN, G. & PARIS, S. (1984). A specific mutation abolishing $Na^+/H^+$ antiport activity in hamster fibroblasts precludes growth at neutral and acidic pH. *Proc. Natl Acad. Sci. USA*, **81**, 4833–4837.

ROTIN, D., STEELE-NORWOOD, D., GRINSTEIN, S. & TANNOCK, I. (1989). Requirement of the $Na^+/H^+$ exchanger for tumor growth. *Cancer Res.*, **49**, 205–211.

ROTIN, D., WAN, P., GRINSTEIN, S. & TANNOCK, I. (1987). Cytotoxicity of compounds that interfere with the regulation of intracellular pH: a potential new class of anticancer drugs. *Cancer Res.*, **47**, 1497–1504.

SPARKS, R.L., POOL, T.B., SMITH, N.K.R. & CAMERON, I.L. (1983). Effects of amiloride on tumor growth and intracellular element contact of tumor cells *in vivo*. *Cancer Res.*, **43**, 73–77.

SUTHERLAND, R.M. (1988). Cell and environment interactions in tumor microregions: the multicell spheroid model. *Science*, **240**, 117–184.

TANNOCK, I.F. & ROTIN, D. (1989). Acid pH in tumors and its potential for therapeutic exploration. *Cancer Res.*, **49**, 4373–4384.

THOMAS, J.A., BUCHSBAUM, R.N., ZIMNIAK, A. & RACKER, E. (1979). Intracellular pH measurements in Erlich ascites tumour cells utilizing spectroscopic probes. *Biochemistry*, **18**, 2210–2218.

THOMSON, J.E. & RAUTH, A.M. (1974). An *in vitro* assay of to measure the viability of KHT tumor cells not previously exposed to culture conditions. *Radiation Res.*, **58**, 262–276.

VAUPEL, P., KALLINOWSKI, F. & OKUNIEFF, P. (1989). Blood flow, oxygen and nutrient supply, and metabolic microenvironment of human tumors: a review. *Cancer Res.*, **49**, 6449–6465.

WIKE-HOOLEY, J.L., HAVEMAN, J. & REINHOLD, J.S. (1984). The relevance of tumour pH to the treatment of malignant disease. *Radiother Oncol.*, **2**, 343–366.